# Ann Chahroudi receives the 2025 ASCI/Marian W. Ropes, MD, Award

**DOI:** 10.1172/JCI199945

**Published:** 2025-12-15

The American Society for Clinical Investigation (ASCI) honors Ann Chahroudi, MD, PhD (Figure 1), with the 2025 ASCI/Marian W. Ropes, MD, Award. Dr. Chahroudi is recognized for her contributions to the development of novel pediatric models of postnatal HIV and Zika virus infections. She is Professor and Vice Chair for Basic Science and Career Development in the Department of Pediatrics at Emory University School of Medicine. Dr. Chahroudi is also Founding Director of the Pediatric Residency Investigative Scholars at Emory program, Codirector of the Emory Center for AIDS Research, and co–principal investigator for the Martin Delaney Pediatric Adolescent Virus Elimination (PAVE) Collaboratory. Dr. Chahroudi was elected to the ASCI in 2022. Dr. Julie Saba, MD, PhD, an ASCI Physician-Scientist Engagement Committee member and The John & Edna Beck Chair in Pediatric Cancer Research at the University of California, San Francisco, interviewed Dr. Chahroudi in July 2025.

Julie Saba: Dr. Chahroudi’s team has used macaque models to identify the sources of persistent infection, to define the anatomical origin of viral rebound, and to test novel cures and approaches. Dr. Chahroudi, how did you come to focus on your area of research?

Ann Chahroudi: My passion centers around HIV research, and it started all the way back in high school. I trace it back to a weekend I spent doing a teen peer AIDS education workshop with the Red Cross. I signed up not understanding a lot about it, but HIV was obviously a big deal in the news in the late ’80s and early ’90s. It still is a major problem, but at that time, there were no effective treatments, and a lot was being learned about the etiology of AIDS as a disease and its different manifestations. It was a scary time to be a teenager and to be thinking about going out in the world with this virus. But what struck me the most, and has stuck with me all these years, is when a man who was living with HIV and had AIDS spoke with us. Hearing his perspective and what he was facing was very powerful. That’s what set me on the trajectory to study HIV. I initially was thinking I was going focus on the vaccine side of things and have moved into cure as the years have evolved. But it still has a great impact on me when we talk with our community advisory board members or other people who are affected by HIV — whether they had a parent with HIV or are living with it themselves. That is the main driver of my passion for this research.

JS: What factors do you feel were most critical to your success in your own research?

AC: Some of it is personal, and some is [the interaction of] internal versus external factors. To be successful in research, you have to work hard, obviously, be determined, and persevere in the face of failure — of which there is a lot in research. I think about the number of grants I submitted to the NIH in my first few years as a junior faculty member, and the number that I got initially was zero for a few years. Physician-scientists are used to being the cream of the crop. When they get to that point [in their career], some keep plugging along, keep submitting, revising, and getting all the feedback they can. Once they break through, it seems like the world opens up. Other people get intimidated or give up a little too early or maybe have a different career path that fits their passion. So, [a key is to be] determined, persevering, and not giving up when you initially have a number of setbacks.

Externally, when I think back to those years, what saved me, what [taught me] a lot and informed my research questions was being part of large teams of researchers who were doing program project grants like the PAVE Collaboratory, which I’m now coleading. You’re exposed to people from different institutions and perspectives, and you begin to build your network. That team-science aspect is important for scientists at all levels, but particularly for junior scientists who are learning how to conduct their research programs — of course, you need to be funded to do so — and to pull out the most interesting and important questions. Those are the determination and team-science approaches. While I was not getting all the grants that I submitted, Emory had a great pilot awards [program]. I applied to nearly every single one and was much more successful with those. And when doing nonhuman primate research, individual pilot awards are often not sufficient to fund the study. Cobbling together funds from different resources was a critical way to get the work done. Finally, I had great support from my division and my department at the beginning: a startup package that I was able to utilize for my lab, for technicians, for equipment. Today I’m seeing that is less and less common. Of course, financial resources now more than ever are constrained, but you have to invest in those individuals early on. It sets them up for success a lot more than [following the K12-K08-R01 sequence] and then getting the resources. I think that’s much too late.

JS: Where do you see your career going now, and can you make some predictions about what might happen in HIV work and clinical opportunities and treatments?

AC: We’re talking in a time of great upheaval and change, although it’s unclear how long this change will be in effect. I doubt things will completely return to what we would probably call normal, but I think there will be some stabilization. I’m encouraging all our trainees and junior faculty to push forward, pursue what they’re passionate about. Research will continue in the US, and it will still be exciting to be a scientist. Looking to the future in my field, we’ve mostly been focusing on studying cure approaches in nonhuman primates, in adults, and, more recently, much more in infant models. We’re not only testing interventions that have been already tried in adults, but trying to learn as much as we can about the developing immune system and how we might be able to specifically target persistent virus in the setting of the infant immune system and [understand] how virus persistence can differ for a child or an infant compared to an adult. Long-acting antiretroviral therapy is still being rolled out and tested for kids, and the question of how available it will be on an international scale [remains]. The group of scientists I have been working with have been pursuing gene therapy for HIV control for many years, as part of the PAVE–Martin Delaney Collaboratory. Some of the research in nonhuman primates has been astounding, and it seems this may be an approach that actually works better in infants than it does in adults, and that likely relates back to the more tolerant immune system. So our team is trying to move slowly but surely towards a clinical trial. I’ll be fascinated to see the outcome and how that might change the landscape of treatment for pediatric HIV.

JS: Could you share your thoughts about the unique challenges and some advantages of working with pediatric populations and pediatric models when studying infectious disease and immunology problems?

AC: It can be challenging when you want to do a big assessment of the immunology and you’re dealing with a very tiny amount of blood and a small number of PBMCs, for example. From a clinical perspective, the first time I did my pediatrics rotation is when I knew I wanted to be a pediatrician. I hadn’t gone into medical school thinking that, but I wondered, “Why would anyone want to do anything else?” as soon as I started interacting with kids and their families. So it made sense to focus my research in that area as well. I think that’s a lesson for everybody who is becoming a physician-scientist. The closer you can link your clinical practice to your research, the better off you will be in the long run. You always think about your mission and understand the “why.”

But to get back to your question, it’s fascinating to be thinking about the immunology and how it changes. I’m not a specialist in the maternal-fetal interface, but everything I learn about it is amazing. From what’s going on in utero to the neonatal period to the development of the immune system in infancy and into childhood — I think that that is so ripe for discovery. And to be able to do the longitudinal studies in the infant nonhuman primate model and ideally translate those into the human setting: that is such a fantastic opportunity. We haven’t done as many studies in my lab with human samples, but that’s an area that we are working on in a bigger group and hoping to do more of in the future. There’s always more to learn, and any time you answer one question, it leads to ten more that we want to go after. I’ve been interested in exploring, and I think a lot of people are, how to break out of the NIH-only funding model and whether there are industry funders or foundations that are interested in this area. That’s where thinking more broadly than just the single pathogen can be effective in terms of looking toward the future. It’s something that we keep talking about — the pivot — in my institution, and that I think is important. But we also need to recognize that some areas of science are more ripe for pivoting and have built longer-term relationships with certain foundations or those interested in funding research. I ask anyone [reading] this from industry or foundations to please think about how you can interact with your pediatrician-scientist colleagues to support the important research, whether it’s on HIV or cystic fibrosis or sickle cell or any of the many chronic conditions affecting children. Their support is needed now more than ever.

JS: What lessons have you learned in your own training? And what advice specifically do you have for trainees?

AC: I’ve learned a lot over the years both in training and while climbing the faculty ranks. A couple of things I’ll mention: first, not to wait to have your personal life. Follow the path that it needs to follow at the same time as your career is following its path. People always ask me, “What’s the right time to have kids? What’s the right time to do this or that?” My answer is always, “The right time is when it’s right for you and your colleagues, and your career can mold itself around that.” And there were times when I felt like I really couldn’t be open about my family. Of course, when you’re in training, you’re not as much in control of your own time. But the physician-scientist aspect of it really did facilitate more freedom and flexibility.

Another [lesson] is that being a physician-scientist opens you up to a wealth of opportunities that come your way because of the dual nature of understanding the research world and the clinical world. It’s very fashionable now to have conversations about how to say no and turn things down. But I usually tell people, at least at the start phase of their career, to be open to opportunities and to say yes, because it can change the course of your career and your trajectory. And you might find something that is a passion that you didn’t know about. That’s how you grow as an individual and a clinician, a scientist, a leader. It’s important to do that at a certain stage. Maybe then you start thinking, “What can I let go that I tried out and wasn’t for me? What can I transition to somebody who’s more junior?” Finally, having that physician-scientist identity and role models in that area. I don’t mean just MD-PhDs, but practicing clinicians who have a research program, whether that’s basic, clinical, translational, implementation, etc. But always having [in mind] the “why,” the physician aspect and what your mission is if you’re studying something in the lab. What is that eventually going to lead to in kids? Being entrepreneurial and trying to turn the discoveries into treatments. And I think that’s so critical. It’s not always easy: not every single experiment works, and sometimes that process is long. This goes back to the team-science aspect as well, because it’s pretty rare that somebody these days discovers a molecule, tests it in animals, designs a clinical trial, and then sees it go into patients in the course of their career. Most of us are building off the discoveries of others and trying to carry that ball and pass it along to the next person. But staying involved in that team allows you to see the fruits of your labor wherever you are along that process of translation.

JS: Thank you so much for sharing your perspectives with us.

AC: Thank you.

*The interview has been edited for length and clarity*.

## Figures and Tables

**Figure 1:**
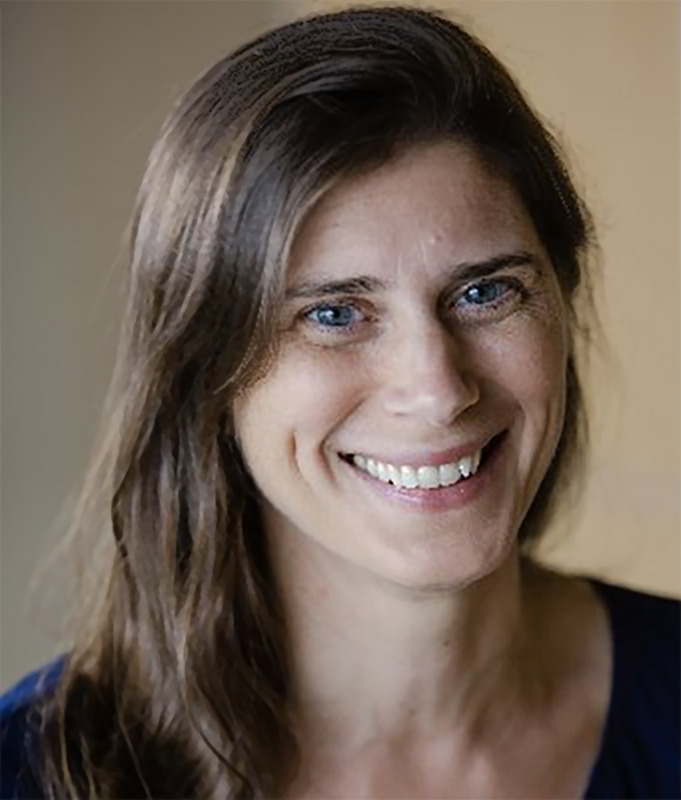
Ann Chahroudi is the recipient of the 2025 Marian W. Ropes Award.

## Version 1 12/15/2025

Electronic publication

